# Experimental study on flow characteristics of gas transport in micro- and nanoscale pores

**DOI:** 10.1038/s41598-019-46430-2

**Published:** 2019-07-15

**Authors:** Weijun Shen, Fuquan Song, Xiao Hu, Genmin Zhu, Weiyao Zhu

**Affiliations:** 1 Key Laboratory for Mechanics in Fluid Solid Coupling Systems, Institute of Mechanics, Chinese Academy of Sciences, Beijing, 100190 China; 2 School of Petrochemical and Energetic Engineering, Zhejiang Ocean University, Zhoushan, 316022 China; 3 School of Civil and Resource Engineering, University of Science and Technology Beijing, Beijing, 100083 China

**Keywords:** Natural gas, Petrol

## Abstract

Gas flow behavior in porous media with micro- and nanoscale pores has always been attracted great attention. Gas transport mechanism in such pores is a complex problem, which includes continuous flow, slip flow and transition flow. In this study, the microtubes of quartz microcapillary and nanopores alumina membrane were used, and the gas flow measurements through the microtubes and nanopores with the diameters ranging from 6.42 μm to 12.5 nm were conducted. The experimental results show that the gas flow characteristics are in rough agreement with the Hagen-Poiseuille (H-P) equation in microscale. However, the flux of gas flow through the nanopores is larger than the H-P equation by more than an order of magnitude, and thus the H-P equation considerably underestimates gas flux. The Knudsen diffusion and slip flow coexist in the nanoscale pores and their contributions to the gas flux increase as the diameter decreases. The slip flow increases with the decrease in diameter, and the slip length decreases with the increase in driving pressure. Furthermore, the experimental gas flow resistance is less than the theoretical value in the nanopores and the flow resistance decreases along with the decrease in diameter, which explains the phenomenon of flux increase and the occurrence of a considerable slip length in nanoscale. These results can provide insights into a better understanding of gas flow in micro- and nanoscale pores and enable us to exactly predict and actively control gas slip.

## Introduction

Fluid flow behavior in the micro- and nanoscale channels and pores is crucial for the development of microelectromechanical systems (MEMS) and nanoelectromechanical systems (NEMS)^1^ and for studies on cells and nanofilms^2^, particularly shale gas resources^3^. Shale gas resources have attracted wide attention and have gradually become an important role in global energy supply, and the pore in shale is less than 50 nm^4,5^. In nanoscale confined gas flows, the gas-wall molecular interactions dominate flow characteristics^6^. These flows demonstrate substantially different physics from continuum descriptions due to rarefaction, surface force field and surface adsorption^7^. Multiple gas transport mechanisms coexist in shale gas reservoirs due to the ultra-fine pore structure. Rarefied gas transport mechanisms in the micro- and nanoscale are further subdivided into slip flow (0.001 < *K*_*n*_ < 0.1) and transition flow (0.1 < *K*_*n*_ < 10)^8–10^. Flow characteristics in the nanoscale pores result in considerable challenges in the study of fluid flow behavior^11–14^. Thus the fundamental understanding of gas flow in the micro- and nanoscale channels and pores is essential to predict the flow characteristics of transition flow and slip flow.

Nanofluid flows have some unique features that are quite different from fluid flow in the conventional scale^15–19^, which cannot be predicted by conventional fluid mechanics. In the past years, the fluid flow in the nanopores has always attracted considerable public concern due to the development in nanofluidic devices and systems. Beskok and Karniadakis^20^ proposed a model for rarefied gas flows in channels, pipes and ducts with smooth surface, which was used to represent the velocity distribution in channels and pipes. Holt *et al*.^21^ found that the flux was considerably larger than the predictions of the H-P equation by as much as three to four orders of magnitude and slip length was up to 3–68 μm, when water and gas flowed through carbon nanotubes membrane with an aperture of 1.3–2 nm. Majumder *et al*.^22^ thought that the magnitude of gas fluxes was 15-fold to 30-fold higher than that predicted from Knudsen diffusion in a carbon nanotubes membrane with the diameter of 7 nm. King^23^ considered that the major effect of wall slip at low oscillation frequencies was the transformation of instantaneous velocity profiles from Poiseuille-like to plug-like, with an overall enhancement of fluid velocity magnitude when gas flowed through a circular nanotube. Barisk and Beskok^6,24^ used the molecular dynamic simulation method to investigate gas flow in nanotubes, and they deemed that density exhibited a universal behavior within the wall force penetration region under different flow conditions. Although the apertures of the hydrophobic carbon nanotubes were extremely small, the flow characteristics between the hydrophobic and hydrophilic nanotubes dramatically differed. Especially the use of hydrophilic nanotubes has limited application in shale nanopores, viruses, bacteria and MEMS/NEMS^25–27^. In addition, the artificial hydrophilic nanotubes is an extremely difficult process. In the recent years, Polymicro Technologies introduced their latest technology for hydrophilic nanocapillary production and the diameter of the smallest nanocapillary was only 200 nm^28^. Wu *et al*.^29^ used a nanofluidic chip with a dimension of 100 nm (depth) × 5 μm (width) × 200 μm (length) to demonstrate flow behavior in nanoscale, but they disregarded flow rate characteristics. And there is still a lack of studies on hydrophilic nanoscale with the diameters ranging from 10 nm to 200 nm because it is extremely challenging to measure flow rate in a single nanotube^30–32^. With the rapid development of technology, the alumina membrane with numerous nanoscale pores emerges gradually. Koklu *et al*.^33^ used an alumina membrane to drive liquid to flow in the nanoscale pores, and the total flux can be easily measured due to the existence of numerous nanopores with the diameter of less than 200 nm in the hydrophilic membrane. The alumina membrane can withstand large pressure gradients because it exhibits high thermal stability and chemical stability^34,35^, and they are preferred due to their well-defined pore geometries^36^. Therefore, there is a good possibility to understand multiple gas transport mechanisms in the micro- and nanoscale channels and pores using the alumina membrane.

In this study, the microtubes of quartz microcapillary and the nanopores of alumina membrane were used, and the gas flow measurements through the microtubes and nanopores with the diameters range of 6.42 μm to 12.5 nm were conducted to study gas flow characteristics. The validity of the H-P equation in the nanopores was analyzed, and then the Knudsen diffusion and slip flow were compared with the experimental results to further analyze the effect of gas slippage in the nanopores. Furthermore, the flow resistance was calculated to explain the increase in flux and the occurrence of an enormous slip length in nanoscale. These results can provide a better understanding of gas flow characteristics in the micro- and nanoscale channels and pores.

## Experimental Materials and Methods

### Experimental materials

**Figure 1:**
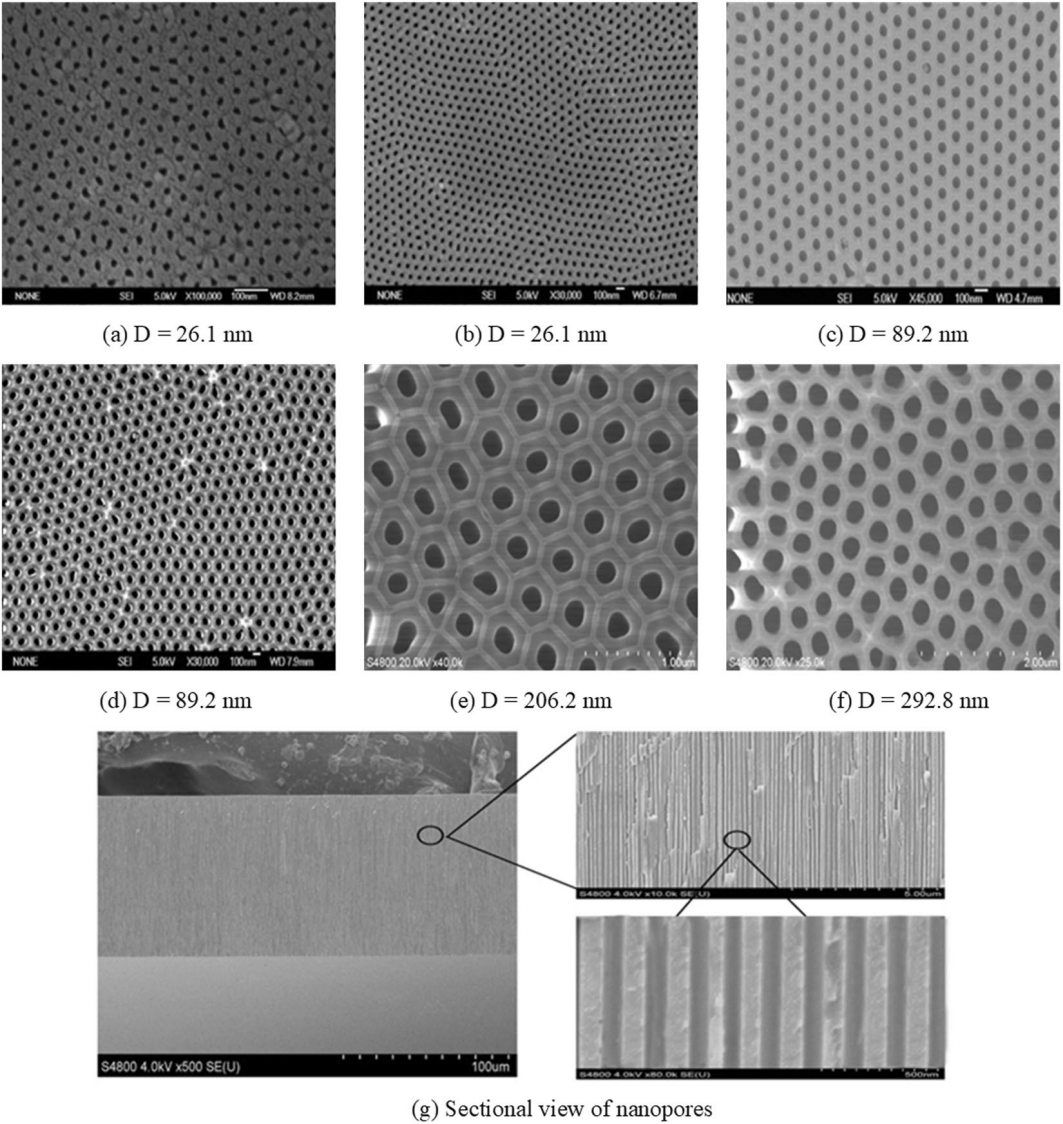
Scanning electron microscope (SEM) images of alumina membranes.

In this study, the microtubes of quartz microcapillary and nanopores alumina membrane were used to study fluid flow behavior in the micro- and nanoscale channels and pores. When gas was driven through the microtubes, the jigs could be moved away and the microtubes would be directly connected to the capillary of the flowmeter. The quartz microcapillary was provided by Polymicro Technologies while the experimental alumina membranes were obtained from Shenzhen Tuopu, which were shown in Fig. 1. As can be illustrated from the Fig. 1, we can see that there are numerous nanopores in the alumina membrane and the sectional view of the nanopores. The detailed measurement of the nanopores are shown in the Supplementary Material (Section S-I).

The diameter and quantity of the nanopores were averaged by the measurements with the Sirion 200 scanning electron microscope, Nanomeasurer and ImageJ software^37^, and the diameter of the microcapillary was measured in our previous work^17^. The characteristics of the microtubes and nanopores are summarized in Table 1. The length-diameter ratio ranges from 301 to 3589, and thus the nanopores of alumina membrane can be considered as the experimental channel under such as a large ratio.

**Table 1:** Some measured characteristics of microtubes and nanopores in this study.

Material	Diameter	Porosity (%)	Quantity (N)/a	Pore length	Length-diameter ratio
quartz microcapillary	6.42 μm	100	1	4.51 cm	7025
quartz microcapillary	14.50 μm	100	1	6.54 cm	4507
alumina membrane	12.50 nm	8.30	2.1 × 10^11^	45.0 μm	3589
alumina membrane	26.10 nm	22.20	1.3 × 10^11^	56.4 μm	2165
alumina membrane	67.00 nm	26.10	2.3 × 10^10^	88.1 μm	1315
alumina membrane	89.20 nm	31.70	1.6 × 10^10^	93.7 μm	1051
alumina membrane	206.20 nm	31.40	3.0 × 10^9^	56.4 μm	274
alumina membrane	292.80 nm	39.30	1.8 × 10^9^	88.1 μm	301

### Experimental methods

**Figure 2:**
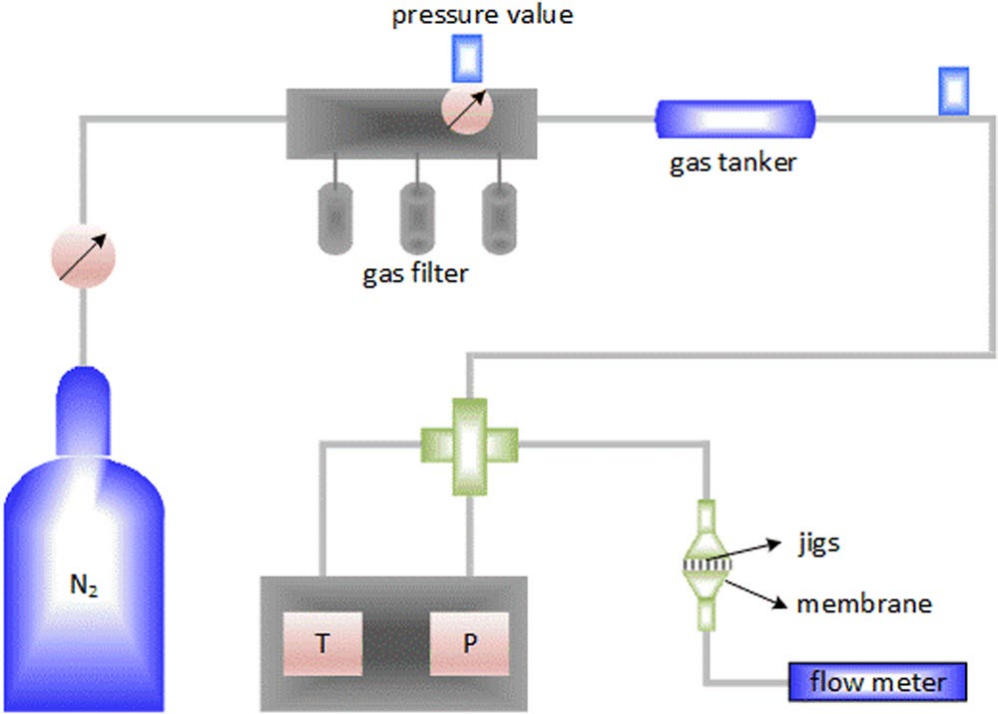
Schematic diagram for apparatus used to measure gas flow in micro- and nanoscale pores.

In this study, gas flow experiments of microtubes and nanopores were conducted to analyze flow characteristics of multiple gas transport mechanisms in the micro- and nanoscale channels and pores. The schematic diagram for apparatus used to measure gas flow in micro- and nanoscale pores was shown in Fig. 2, which was composed of nitrogen (N_2_) gas source, gas filter, gas tanker, flow meter, temperature and pressure measuring system. The experiments were performed in a customized equipment of VS-840U clean bench, and the temperature maintained at 20 °C. The various parts of the equipment can be connected by a plastic hose, which can bear high pressure. The experimental membrane was first placed in the jigs with sandstone support, and the jigs and membrane were tightly sealed with two sealing clamps and two sealing rubber circles. And the high-purity nitrogen gas was sterilized in a gas filter and stored in a gas tank. Then the nitrogen was driven through the experimental nanopores under 0.01 to 0.2 MPa using the pressure valve. Finally, the flux was directly measured using an MF4000 series electronic micro flowmeter. In the experiment, temperature and pressure were measured with the thermometer and pressure gauge, respectively. The experiments were repeated thrice using different alumina membranes, and the experimental data exhibited high reproducibility.

## Results and Discussion

### H-P equation

In the micro- and nanoscale channels and pores fluid flow is in a laminar flow state^38,39^ and the Hagen-Poiseuille (H-P) equation can be used to describe the theoretical gas flux. And it may be expressed as

1 $${Q}_{{\rm{h}}p}=\pi {D}^{4}pM(dp/dl)/(128{\mu }_{g}ZRT)$$

where *Q*_*hp*_ is the theoretical gas flux; *D* is the diameter of the nanopores and microtubes; *p* is the pressure; *M* is the gas molar mass; *μ*_g_ is the gas viscosity; *Z* is the gas deviation factor; *R* is the universal gas constant; *T* is the temperature; d*p/*d*l* is the pressure gradient; and *l* is the length of the pore.

In accordance with the experimental results from the gas flowmeter under different driving pressures, the experimental flux of a single microtube and nanopore can be obtained by the following form

2 $${Q}_{\exp }={Q}_{{\rm{m}}}/N$$

where *Q*_exp_ is the experimental flux of a single microtube and nanopore; *Q*_m_ is the total flux measured by the gas flowmeter; *N* is the quantity of pores, which is shown in Table 1.

**Figure 3:**
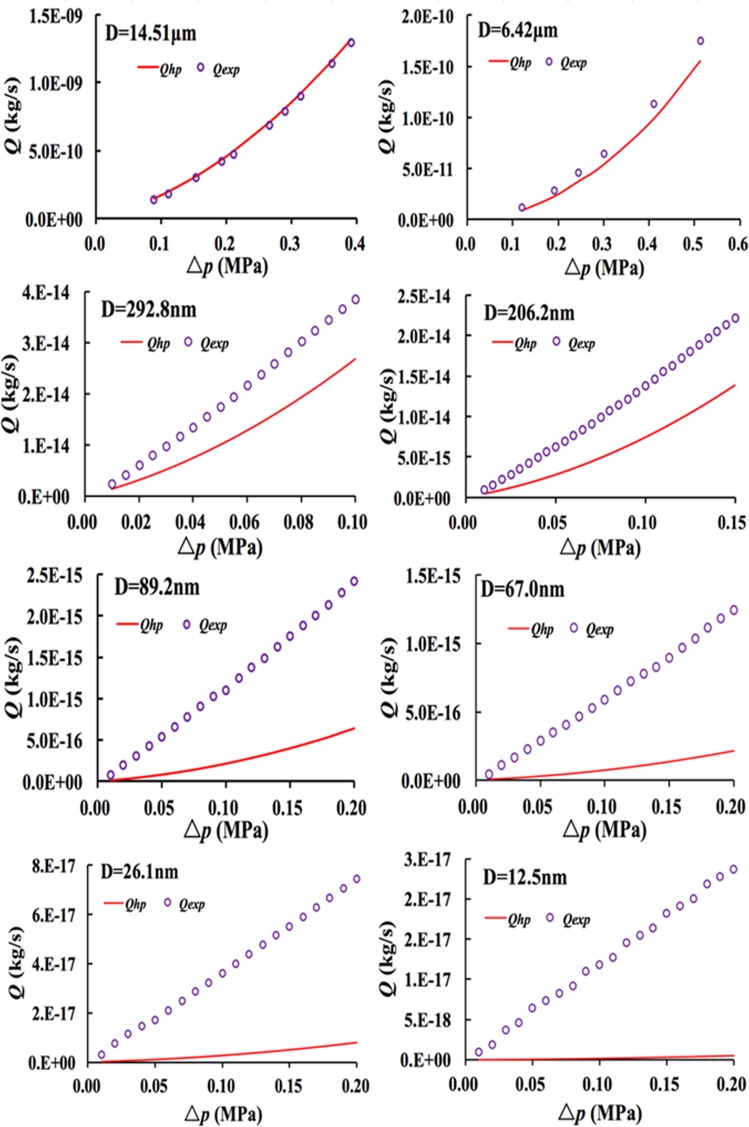
Comparison of experimental gas flux and H-P flux.

The experimental flux and theoretical gas flux calculated by the H-P equation in micro- and nanoscale pores are shown in Fig. 3. As can be seen from the Fig. 3, the experimental gas flux in micro- and nanoscale pores have similar variation characteristics. With the increase of the pressure gradient, the experiment gas flux increases approximately linearly. The experimental flux agrees well with that predicted by the H-P equation when gas flows through the quartz microcapillary with the diameter of 14.51 μm, whereas the experimental flux slightly increases when the diameter decreases to 6.42 μm. It can be illustrated from the Fig. 3 that the deviations between the experimental flux and theoretical flux will increase considerably with the decrease of the diameter ranging from 292.8 nm, 206.2 nm, 89.2 nm, 67.0 nm, 26.1 nm to 12.5 nm. It implies that a small nanopore diameter will lead to high deviation between the experimental flux and theoretical flux. The experiment flux of gas flow through the nanopores is higher than that predicted by the H-P equation by one order of magnitude (or two), and the H-P equation considerably underestimates gas flux in the nanoscale pores. Therefore, the transition flow characteristics (0.1 < *K*_*n*_ < 10) should be considered when the diameter of the nanopores range from 12.5 nm to 292.8 nm, and the Knudsen flow, slip flow, slip length and flow resistance in the nanopores need be analyzed to explain the aforementioned phenomenon.

### Knudsen diffusion

When *K*_*n*_ exceeds 10, the gas transport in the nanopores is the Knudsen diffusion^40–43^. The regime is controlled by Knudsen diffusion, and it may be expressed as

3 $${K}_{n}={\lambda }/D$$

where *K*_*n*_ is the Knudsen number for real gas; $\lambda$ is the mean free path of the molecules for real gas, $\lambda =(\mu \sqrt{\pi ZRT/2M})/P$.

Table 2 indicates that the gas is in the transient flow (slip flow) area when gas flows through the microtubes and nanopores. Bulk gas transport mechanisms in the nanopores include viscous flow, slip flow and transition flow, and slip flow and Knudsen diffusion are two different gas transport mechanisms^9,44,45^. The slip flow takes place when the intermolecular collision dominates, and the Knudsen diffusion occurs when the collision between gas molecule and nanopore wall is dominant. In the nanopores, the slip flow and Knudsen diffusion coexist in gas transport mechanisms under a certain pressure and temperature condition. Thus, it is necessary to characterize the share of each transport mechanism in the total transport. The coupling between the two types of diffusion is not considered because the Javadpour model is a linear sum of slip flow and Knudsen diffusion^46^. The model developed by Anderson *et al*.^47^ also describes all transport mechanisms, but it includes the empirical coefficients determined by experimental data. Wu *et al*.^14^ selected the frequency ratios of collisions between the molecules and the gas-wall molecules to the total collision frequency as the weighting factors of slip flow and Knudsen diffusion to describe flow characteristics in the nanopores. Therefore, in accordance with the aforementioned principle^14^, the flow rate of Knudsen diffusion in the nanopores can be expressed as

4 $${Q}_{{Kn}}=\frac{2}{3}N\pi \frac{{D}^{3}}{8}{\delta }^{{D}_{{\rm{f}}}-2}{(\frac{8ZM}{\pi RT})}^{0.5}\frac{{C}_{{\rm{g}}}p}{Z(1+1/Kn)}\frac{{\rm{d}}p}{{\rm{d}}l}$$

where *Q*_*Kn*_ is the flow rate of Knudsen diffusion; *δ* is the ratio of normalized molecule size to local average pore diameter^14^, *δ* = 0.5; *D*_f_ is a quantitative measure of surface roughness that varies between 2 and 3, representing a smooth surface and a space-filling surface, respectively^48^; *C*_g_ is the gas compression factor, *C*_g_ = 1/*p* − d*Z*/(*p* × d*Z*).

**Table 2:** Gas flow mechanisms in the micro- and nanoscale pores.

Parameter	Viscous flow	Slip flow	Transient flow
diameter (*D*)	14.51 μm	6.42 μm	292.8 nm, 206.2 nm, 89.2 nm, 67.0 nm, 26.1 nm and 12.5 nm
Knudsen number (*K*_*n*_)	*K*_*n*_ < 0.001	0.001 < *K*_*n*_ < 0.1	0.1 < *K*_*n*_ < 5

**Figure 4:**
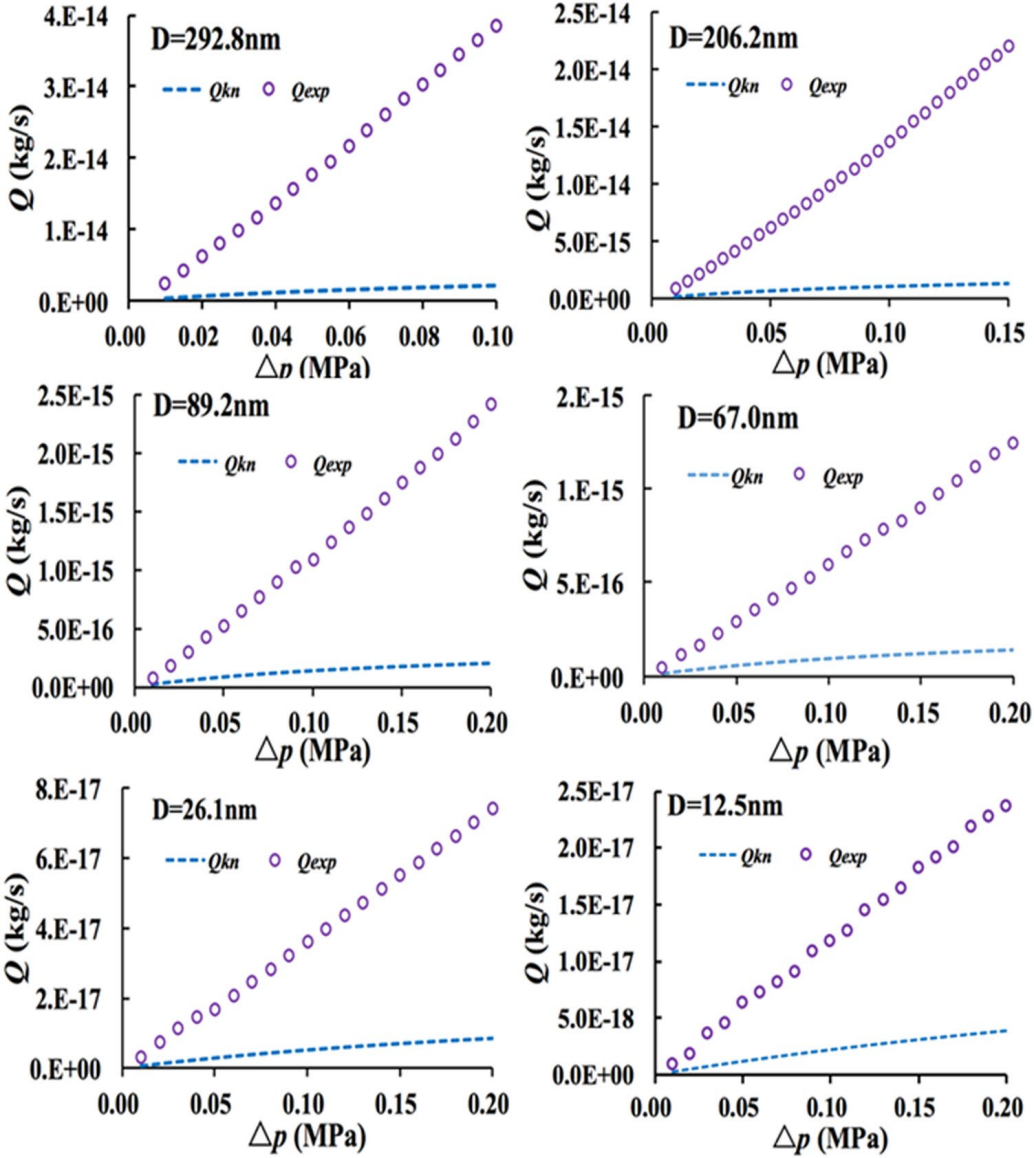
Comparison of the experimental flux and Knudsen diffusion.

The comparison of flow characteristics between the experimental flux and Knudsen diffusion is illustrated in Fig. 4. It can be seen from the Fig. 4 that the experimental flux is larger than the Knudsen diffusion flux, and the deviations between Knudsen diffusion and the experimental flux decrease with the decrease of the diameter. Thereby it implies that the collision frequency between gas molecules and the wall increases, and the contribution of Knudsen diffusion to the experimental flux also increases. As shown in Figs 3 and 4, the H-P equation and Knudsen diffusion equation are smaller than the experimental flux, and thus they cannot depict the experimental flux. Consequently the slip flow in the nanopores should be analyzed further.

### Effect of slip flow

In this study, the experimental flux in the nanoporous can be measured, which is the total gas flux. When slip flow and Knudsen diffusion coexist in the nanopores, the experimental viscous flow flux with slip effect will be obtained by removing the contribution of Knudsen diffusion, which is caused by the positive slip boundary condition. And the real gas slippery flux can be expressed as

5 $${Q}_{{\rm{s}}}={Q}_{\exp }-{Q}_{{\rm{Kn}}}$$

where *Q*_s_ is the real gas slippery flux.

In this study, the gas slippage effect in the nanopores is analyzed by calculating the experimental slip length. The generalized flow boundary condition proposed by Navier can be written as^49^

6 $${v}_{{\rm{s}}}=b{\frac{\partial {v}_{x}}{\partial {\rm{z}}}|}_{{\rm{wa}}ll}$$

where *v*_s_ is the slip velocity, *v*_s_ = *Q*_s_/(*πD*^2^/4); *z* is the coordinate along the normal direction of the interface; *b* is the slip length; *v*_x_ is the tangential velocity in the *x*-axis direction.

**Figure 5:**
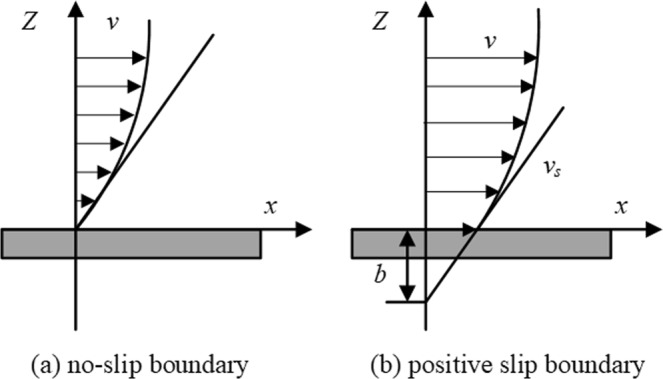
Schematic diagram of no-slip and positive slip boundary.

When gas flows through a macroscale channel, the concept of the slip boundary condition was first illustrated by Navier^49^ and illustrated schematically in Fig. 5.

When considering the slippage effects, the slip length with various diameters are calculated using the Eqs (1) and (7)^50^.

7 $${v}_{\exp }/{v}_{s}=1+8b/D$$

where *v*_exp_ is the experimental velocity, *v*_exp = _*Q*_exp_/(*πD*^2^/4).

**Figure 6:**
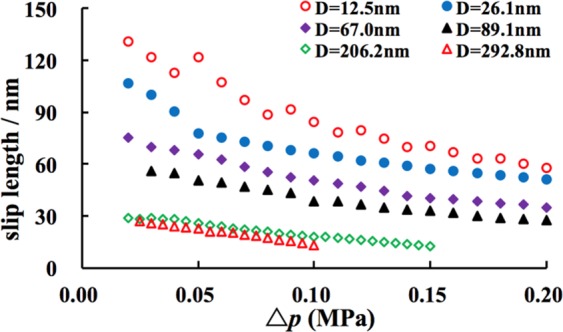
Relationship between slip length and pressure.

The relationships between slip length and driving pressure at different nanopore diameters are shown in Fig. 6. As can be seen from the Fig. 6, a positive slip length exists for gas flow through the hydrophilic nanopores in the alumina membrane, and a small diameter leads to a large slip length. When the diameter of the nanopore is 12.5 nm, the slip length reaches 131 nm under the experimental pressure. It is nearly 11 times the diameter of the nanopore, which indicates that the effect of slip flow increases with the decrease of the diameter. Therefore, the slip length can be disregarded in the macroscale, but it has a considerable effect on the fluid in the nanoscale. This is why that the experimental flux is larger than the H-P equation and Knudsen diffusion due to the significant contribution of slip flow.

Figure 6 also illustrated the change process of slip velocity at the boundary under the experimental pressure from 0 to 0.2 MPa. The slip length decreases with the increase in driving pressure, and several factors contribute to such phenomenon. As the shear rate $(\partial {v}_{{\rm{x}}}/\partial z)$ rapidly increases with the increasing pressure, velocity at the boundary increases under high displacement pressure, and the slope of the gas flow rate (slip length *b*) will decrease with the increase in the pressure. Moreover, the effects of viscous flow and Knudsen diffusion decrease under the high pressure, and the two factors will lead to the decrease in slip length^14^. The slip length results of Qin *et al*.^19^, Holt *et al*.^21^ and Majumdar *et al*.^22^ were all under one atmosphere, and thus they obtained one value of slip length. Our experiments were conducted under different pressures, so the change process of slip length can be obtained in our experiments. Moreover, the gas is located in the transient flow area. The contribution of Knudsen diffusion to the experimental flux was eliminated when gas flowed through the nanopores, and the slip length value is relatively small when the experimental flux is calculated as slip flow.

### Resistance analysis

The gas flow resistance is analyzed to explain the increase in the experimental gas flux and the occurrence of a positive slip length. According to the H-P equation, the relationship between theoretical flow resistance and Reynolds number can be expressed as^17^

8 $$f\times {Re}=64$$

where *Re* is the Reynolds number, *Re* = *ρv*_exp_ *d*/*μ*; *f* is the flow resistance, *f* = 2*D*Δ*p*/(*l ρ v*^2^_exp_).

When the gas is driven through the nanopores, the compressibility should be considered and the experiments should be regarded as a one-dimensional, regular, and equal-sectioned adiabatic frictional compressible flow. The friction coefficient can be expressed by the following form^51^

9 $$f=\frac{D}{4L}\{\frac{1}{\gamma }(\frac{1}{M{a}_{0}^{2}}+\frac{1}{M{a}_{1}^{2}})+\frac{1+\gamma }{2\gamma }(\frac{M{a}_{1}^{2}(2+(\gamma -1)M{a}_{0}^{2})}{M{a}_{0}^{2}(2+(\gamma -1)M{a}_{1}^{2})})\}$$

where *Ma*_0_ is the outlet Mach number; *Ma*_1_ is the inlet Mach number; $\gamma$ is the specific heat ratio, i.e., $\gamma$ = 1.4.

The flow resistance coefficient (C*) is defined to compare the deviation between the experimental resistance and theoretical resistance, which is can be written as

10 $${C}^{\ast }=64/{(f\times \mathrm{Re})}_{\exp }$$

**Figure 7:**
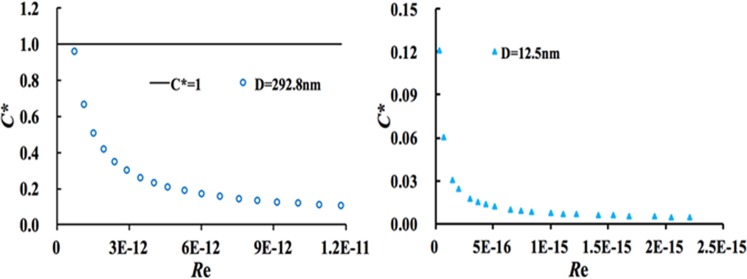
Relationship between resistance coefficient (C*) and Reynolds number (*Re*).

The relationships between resistance coefficient (C*) and experimental Reynolds number (*Re*) are shown in Fig. 7, and other results of the alumina membrane with diameter of 206.2 nm, 89.2 nm, 67.0 nm are illustrated in the Supplementary Material (Section S-II). As can be illustrated from the Fig. 7, the experimental resistance is smaller than the theoretical value in the nanopores. The gas flow resistance rapidly decreases with the increase in driving pressure, and the flow resistance finally becomes constant under high driving pressure. Furthermore, the small nanopores will lead to a small flow resistance, which results in high deviations between the experimental flux and the H-P equation and considerable slip length and slip flow influence. The analysis results about the major head loses rousing from the viscous effects and the minor head loses due to the entrance, area change and the exit in the entire system in the Supplementary Material (Section S-III) show that they are much smaller than the driving pressure in the experimental systems, and will have little effect on the gas flow through the nanopores. The results can explain the gas flow phenomenon in the nanopores that is attributed to a smaller flow resistance than the traditional value in the nanopores. Gas velocity near the wall is higher than zero, which will result in an increase in flux and the occurrence of a considerable positive slip length. The H-P equation fails to depict flow characteristics in the nanopores, and the effect of gas slippage is quite significant.

## Conclusions

In this study, the microtubes of quartz microcapillary and nanopores alumina membrane were used, and the gas flow measurements through microtubes and nanopores with diameters ranging from 6.42 μm to 12.5 nm were conducted to gas flow characteristics in micro- and nanoscale pores. The experimental results indicate that the experimental flux agrees well with the H-P equation in the microscale, whereas the flux of gas in the nanopores is larger than that in the H-P equation by more than an order of magnitude. The H-P equation considerably underestimates gas flux with the decrease in the nanoscale diameter, and the Slip flow and Knudsen diffusion coexist in the experimental nanopores. The experimental flux is larger than Knudsen diffusion, and the contribution of Knudsen diffusion to the experimental flux increases with the decrease in the diameter. The effects of gas slippage are considerable, gas velocity near the wall is higher than zero. The slip length effects decrease with the increase in diameter while slip length decreases with increasing driving pressure. Moreover, the experimental resistance is smaller than the theoretical value in the nanopores, and the flow resistance decreases with the decrease of the diameter, which explains the increase in flux and the occurrence of the significant positive slip length in the nanoscale.

## Supplementary information

Supplementary Information
