# Supplementary material for: Experimental study on flow characteristics of gas transport in micro- and nanoscale pores
Source: Sci Rep. 2019 Jul 15;9:10196. doi: 10.1038/s41598-019-46430-2 (PMC6629846; doi:10.1038/s41598-019-46430-2)
Supplement: Supplementary file 1 — Supplementary Information [file 41598_2019_46430_MOESM1_ESM.doc]

**Supplementary Information**

**Experimental study on flow characteristics of gas transport in micro- and nanoscale pores**

Weijun Shen^1^, Fuquan Song^2, *^, Xiao Hu^2^, Genmin Zhu^2^, Weiyao Zhu^3^

^1^ Institute of Mechanics, Chinese Academy of Sciences, Beijing 100190, China

^2^ School of Petrochemical and Energy Engineering, Zhejiang Ocean University, Zhoushan 316022, China

^3^ School of Civil and Environmental Engineering, University of Science and Technology, Beijing 100083, China

^*^ Corresponding author: songfuquan@zjou.edu.cn

**Section S-I: Measurement of Average Pore Diameter and Density**

**
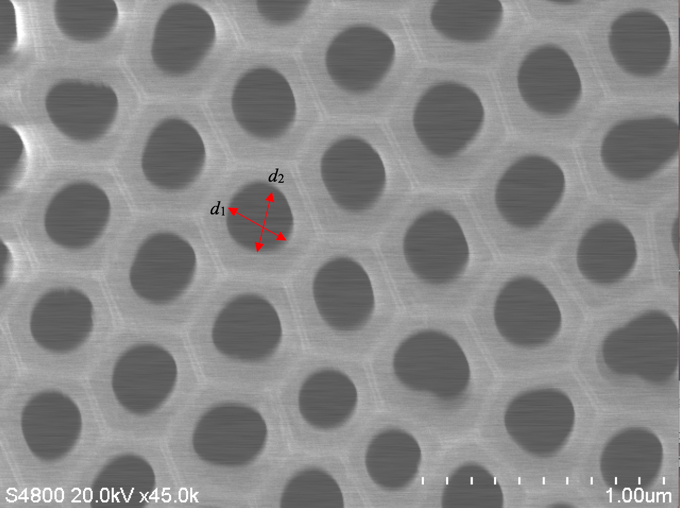
**

Figure. S1. The diameter measured by Nanomeasurer

The diameters were measured by the software two time per pore, which was illustrated in Fig. S1. The results of the diameters measured by Nanomeasurer^1^ were shown in Fig. S2, and the serial number was the number of the nanopore. The diameter of the micro-capillary was measured by our previous paper^2^. All the results were shown in Table 1, and the length to diameter ratio is in the range of 301-3589. Thus, the nanopores of alumina membrane can be taken as the experimental channel under such as a large ratio.


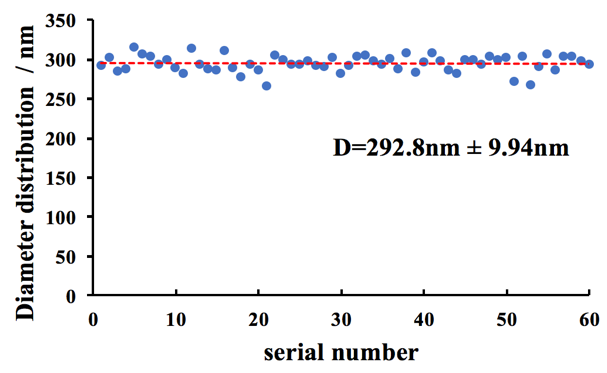

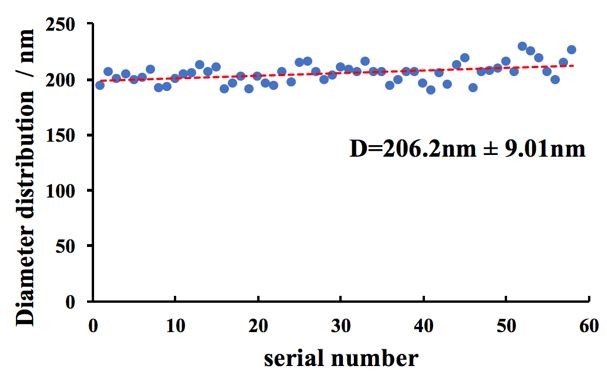


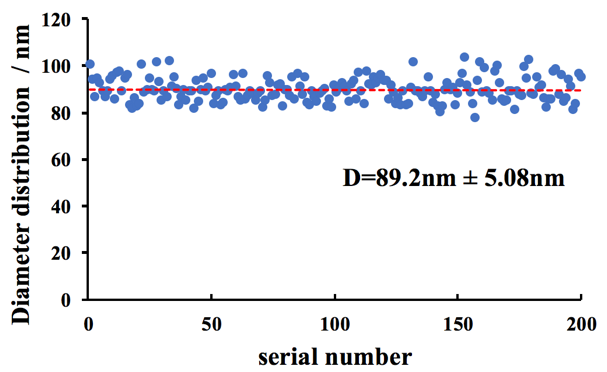

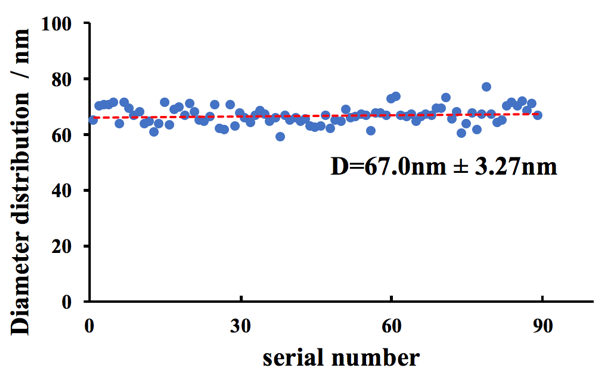


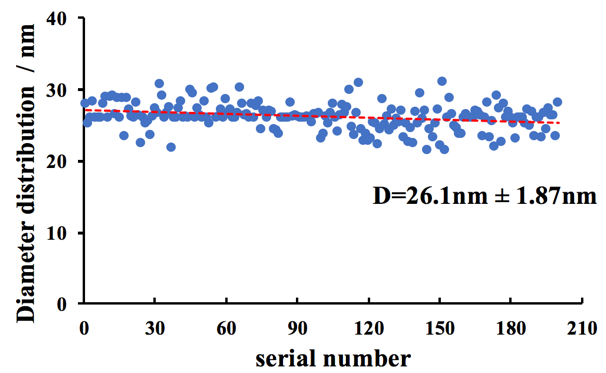

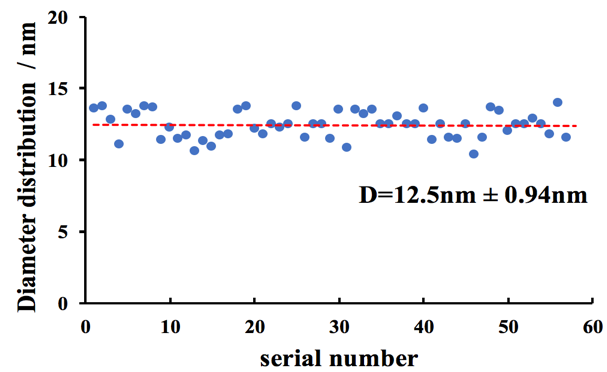


Figure. S2. The results of the diameters in alumina membrane

**Section S-II: Flow Resistance Coefficient of the Alumina Membrane**

The flow resistance coefficients of the alumina membrane were shown in Fig. S3. The flow resistance coefficient was much smaller than the theoretical value in the nanopores.


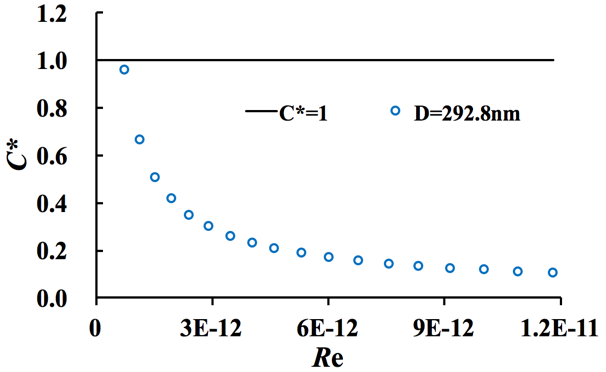

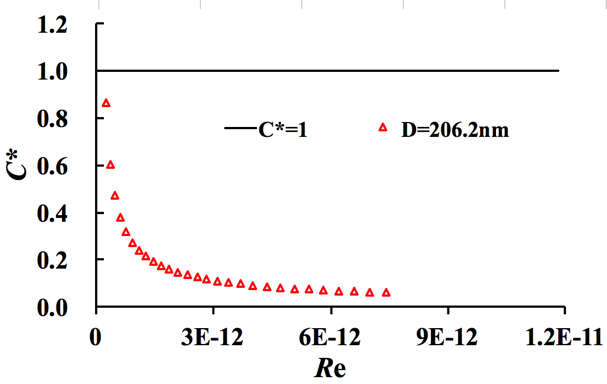


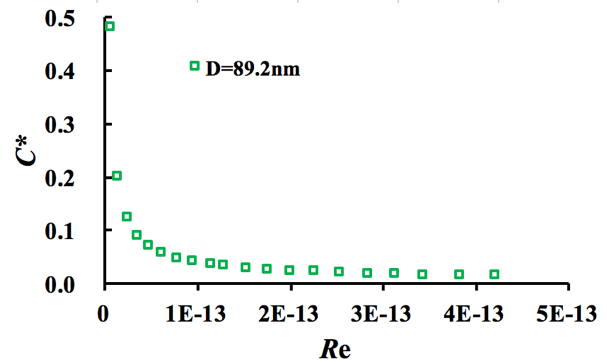

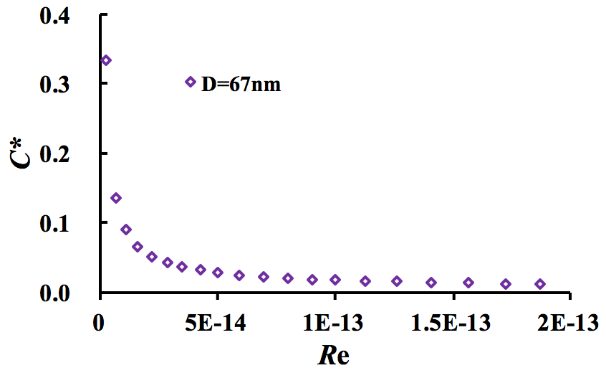

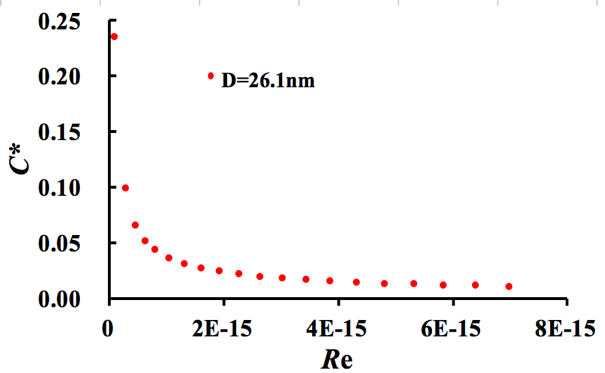

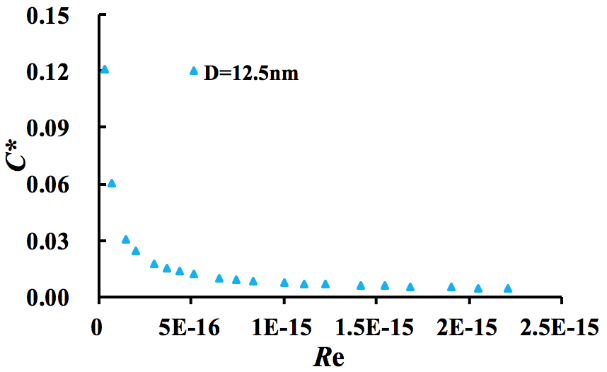


Figure. S3. The relationship between Reynolds number (*Re*) and flow resistance coefficient (C*)

**Section S-III: Extended Bernoulli Analysis**

The extended Bernoulli equation was applied between the pressure gauges, which was expressed as


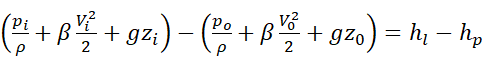
 (S1)

where
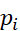
 and
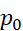
 are the inlet and outlet pressure, respectively;
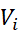
and
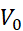
 are the averaged fluid velocities in the inlet and outlet, respectively;
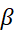
 is the kinetic energy coefficient;
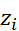
 and
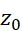
 are the fluid heights in the inlet and outlet, respectively;
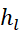
 and
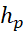
 are the head loss along the entire system and pump head (
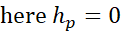
), respectively;
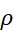
 is the fluid density. The velocities of inlet and outlet are assumed same
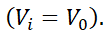
 The fluid height difference between the inlet and outlet is considered as zero
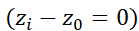
. Therefore, the equation can be simplified into


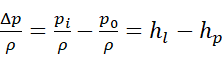
 (S2)

The head loss along the entire system contains two parts: major head losses arising from the viscous effects (
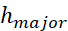
), and minor head loses due to the entrance, area change and exit in the entire system (
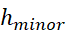
)^3^.The major head loss can be written as


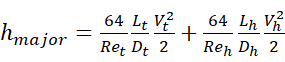
 (S3)

where
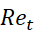
 and
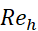
 are the Reynolds numbers in the tube and the membrane holder, respectively;
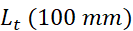
 and
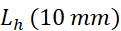
 are the lengths of the tube and the membrane holder, respectively;
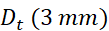
 and
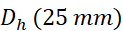
 are the diameters of the tube and the membrane holder, respectively;
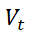
and
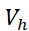
 are the fluid velocities in the tube and the membrane holder, respectively. The minor head loss in the entire system can be expressed as


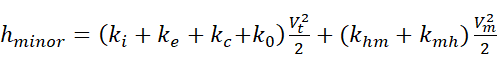
 (S4)


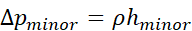
 (S5)

where
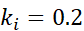
,
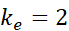
,
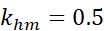
,
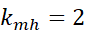
,
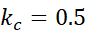
 and
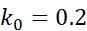
 are the loss coefficients of entrance from the
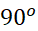
 shoulder to the tube, expansion from the tube to the membrane holder, entrance from the membrane holder to nanopores, exit from the nanopores to the membrane holder, contraction from the membrane holder to the tube, and exit from the tube to the
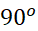
 shoulder tube^4^. Due to the mass continuity, the flow rate through the system is a constant, hence
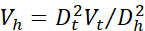
 and
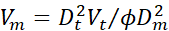
 with
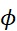
 as the porosity of the membrane and
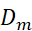
 as the wetted diameter of the membrane^3^.


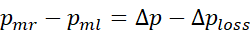
 (S6)


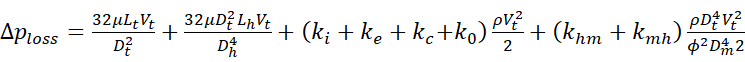
 (S7)

where,
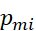
 and
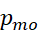
 are the pressures on the inlet side and outlet side of the membrane, respectively. The volumetric flow rate induced by the pressure gradient across the membrane can be written as


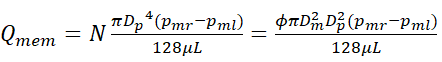
 (S8)

where
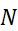
 is the number of pores (
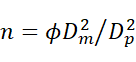
);
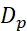
 is the diameter of nanopore and
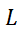
 is the thickness of the membrane. Pressure losses were shown in the Table S1- S6

TABLE. S1. Pressure loss of the nano-membrane in the 292.8 nm pore size

| △*p*(MPa) | *Q*_mem_(m^3^/s) | *V*_t_(m/s) | *h*_major_(MPa) | *h*_minor_(MPa) | △*p*_loss_(MPa) |
| --- | --- | --- | --- | --- | --- |
| 0.01 | 4.45×10^-6^ | 0.55 | 3.44×10^-6^ | 5.00×10^-7^ | 3.94×10^-6^ |
| 0.05 | 3.23×10^-5^ | 3.98 | 2.50×10^-5^ | 2.64×10^-5^ | 5.15×10^-5^ |
| 0.075 | 5.19×10^-5^ | 6.40 | 4.02×10^-5^ | 6.82×10^-5^ | 1.09×10^-4^ |
| 0.10 | 7.08×10^-5^ | 8.74 | 5.48×10^-5^ | 1.27×10^-4^ | 1.82×10^-4^ |

TABLE. S2. Pressure loss of the nano-membrane in the 202.6 nm pore size

| △*p*(MPa) | *Q*_mem_(m^3^/s) | *V*_t_(m/s) | *h*_major_(MPa) | *h*_minor_(MPa) | △*p*_loss_(MPa) |
| --- | --- | --- | --- | --- | --- |
| 0.01 | 2.47×10^-6^ | 0.35 | 2.20×10^-6^ | 2.04E-07 | 2.40×10^-6^ |
| 0.05 | 1.61×10^-5^ | 2.28 | 1.43×10^-5^ | 8.67×10^-6^ | 2.30×10^-5^ |
| 0.10 | 3.54×10^-5^ | 5.01 | 3.15×10^-5^ | 4.18×10^-5^ | 7.34×10^-5^ |
| 0.15 | 5.70×10^-5^ | 8.06 | 5.06×10^-5^ | 1.08×10^-4^ | 1.59×10^-4^ |

TABLE. S3. Pressure loss of the nano-membrane in the 89.2 nm pore size

| △*p*(MPa) | *Q*_mem_(m^3^/s) | *V*_t_(m/s) | *h*_major_(MPa) | *h*_minor_(MPa) | △*p*_loss_(MPa) |
| --- | --- | --- | --- | --- | --- |
| 0.01 | 1.11×10^-6^ | 0.16 | 9.86×10^-7^ | 4.11×10^-8^ | 1.03×10^-6^ |
| 0.10 | 1.52×10^-5^ | 2.16 | 1.35×10^-5^ | 7.74×10^-6^ | 2.13×10^-5^ |
| 0.15 | 2.43×10^-5^ | 3.45 | 2.16×10^-5^ | 1.98×10^-5^ | 4.15×10^-5^ |
| 0.20 | 3.36×10^-5^ | 4.76 | 2.99×10^-5^ | 3.77×10^-5^ | 6.77×10^-5^ |

TABLE. S4. Pressure loss of the nano-membrane in the 67.0 nm pore size

| △*p*(MPa) | *Q*_mem_(m^3^/s) | *V*_t_(m/s) | *h*_major_(MPa) | *h*_minor_(MPa) | △*p*_loss_(MPa) |
| --- | --- | --- | --- | --- | --- |
| 0.01 | 9.45×10^-7^ | 0.13 | 8.40×10^-7^ | 2.98×10^-8^ | 8.69×10^-7^ |
| 0.10 | 1.26×10^-5^ | 1.78 | 1.12×10^-5^ | 5.29×10^-6^ | 1.65×10^-5^ |
| 0.15 | 1.92×10^-5^ | 2.71 | 1.70×10^-5^ | 1.22×10^-5^ | 2.93×10^-5^ |
| 0.20 | 2.66×10^-5^ | 3.77 | 2.37×10^-5^ | 2.36×10^-5^ | 4.74×10^-5^ |

TABLE. S5. Pressure loss of the nano-membrane in the 26.1 nm pore size

| △*p*(MPa) | *Q*_mem_(m^3^/s) | *V*_t_(m/s) | *h*_major_(MPa) | *h*_minor_(MPa) | △*p*_loss_(MPa) |
| --- | --- | --- | --- | --- | --- |
| 0.01 | 2.79×10^-7^ | 0.04 | 2.47×10^-7^ | 2.59×10^-9^ | 2.50×10^-7^ |
| 0.10 | 3.17×10^-6^ | 0.45 | 2.82×10^-6^ | 3.36×10^-7^ | 3.16×10^-6^ |
| 0.15 | 4.84×10^-6^ | 0.69 | 4.30×10^-6^ | 7.82×10^-7^ | 5.09×10^-6^ |
| 0.20 | 6.52×10^-6^ | 0.92 | 5.79×10^-6^ | 1.41×10^-6^ | 7.21×10^-6^ |

TABLE. S6. Pressure loss of the nano-membrane in the 12.5 nm pore size

| △*p*(MPa) | *Q*_mem_(m^3^/s) | *V*_t_(m/s) | *h*_major_(MPa) | *h*_minor_(MPa) | △*p*_loss_(MPa) |
| --- | --- | --- | --- | --- | --- |
| 0.01 | 1.67×10^-7^ | 0.02 | 1.48×10^-7^ | 9.26×10^-10^ | 1.49×10^-7^ |
| 0.10 | 2.17×10^-6^ | 0.31 | 1.92×10^-6^ | 1.56×10^-7^ | 2.08×10^-6^ |
| 0.15 | 3.33×10^-6^ | 0.47 | 2.96×10^-6^ | 3.70×10^-7^ | 3.33×10^-6^ |
| 0.20 | 4.33×10^-6^ | 0.61 | 3.85×10^-6^ | 6.26×10^-7^ | 4.48×10^-6^ |

All of those facts showed that the major head loses rousing from the viscous effects and the minor head loses due to the entrance, area change and the exit in the entire system were much smaller than the driving pressure in the experimental systems, which have little effect on the gas flow through the nanopores.

**Section S-IV: Calculation of the Mass Flow Rate**

It was necessary to consider the compressibility of gas under different pressure. According to the principle of mass conservation, the mass flowrate was equal in the same channel, and the mass flowrate was used in Equation (1). The derivation of Equation (1) was as follow


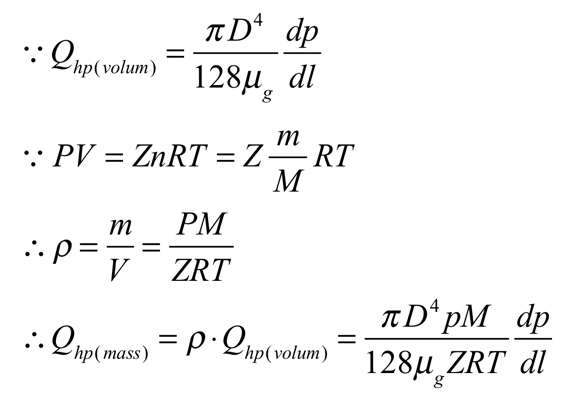
 (S9)

The tube diameter and nanotube length are all under the same certain experimental conditions, and the only variation is pressure. Thus, the x axis is described as Δ*p*.

# References

1. Whitby, M., Cagnon, L., Thanou, M., et al. Enhanced fluid flow through nanoscale carbon pipes. Nano Letters. **8**, 2632-2637 (2008).
2. Song, F.Q., Jiang, R.J., Bian, S.L. Measurement of threshold pressure gradient of microchannels by static method. Chinese Physics Letters. **24**, 1995-1998 (2007).
3. Koklu, A., Li, J., Sengor, S., et al. Pressure-driven water flow through hydrophilic alumina nanomembranes. Microfluidics and Nanofluidics. **21**, 124 (2017).
4. Munson, B.R., Young, D.F., Okiishi, T.H. Fundamentals of Fluid Mechanics. John Wiley and Sons, Inc. 3ed. (1998).
